# RACIAL REPRESENTATIVENESS AND AFFORDABLE HOUSING DISPARITIES: A NATIONAL STUDY OF NURSING ASSISTANTS

**DOI:** 10.1093/geroni/igad104.3551

**Published:** 2023-12-21

**Authors:** Sarah Holmes, Merve Gurlu, Shijun Zhu, Briana Murray, Michael Lepore

**Affiliations:** University of Maryland School of Nursing, Baltimore, Maryland, United States; University of Maryland, Baltimore, Baltimore, Maryland, United States; University of Maryland School of Nursing, Baltimore, Maryland, United States; University of Maryland School of Nursing, Baltimore, Maryland, United States; University of Maryland School of Nursing, Baltimore, Maryland, United States

## Abstract

Occupational racial representation is increasingly recognized as a health equity issue. In the US, racial unrepresentativeness in the healthcare workforce, including nursing homes (NHs), is a pervasive aspect of economic inequality: racially minoritized women are disproportionately overrepresented among nursing assistants (NAs) in NHs, and racially minoritized NAs commonly live in poverty and lack affordable housing. Despite evidence of associations between occupational racial representation and affordable housing, there is limited research on these associations among NAs in NHs. We examined how state-level racial representativeness of NAs in NHs relates to housing affordability using data from the PHI Workforce Data Center, LTCFocus, and US Census. Racial representativeness was calculated as the difference between the percentage of racial group (Black, White) in NA workforce in the state and the percentage of racial group in state working-age population. Multivariable linear regression was used to examine associations between housing affordability and racial representation. The national sample included 527,480 NAs. Results showed overrepresentation of Black NAs (M=17.5%, SD=.166) and underrepresentation of White NAs (M=-18.2%, SD=.127) in most states. Controlling for NH primary payer source and NA education level, lack of affordable housing was associated with higher representation of Black NAs in the workforce (b=1.78, p<.001, R2=.66) and lower representation of White NAs in the workforce (b=-1.45, p<.001, R2=.61). These findings indicate racial disparities in NA access to affordable housing and highlight the need for additional research on these associations and related outcomes to develop equity-centered strategies in the NH workforce that will address housing affordability.

